# Postictal psychosis concealed during long‐term video‐electroencephalographic monitoring and unmasked after discharge: A case report

**DOI:** 10.1002/pcn5.70321

**Published:** 2026-03-20

**Authors:** Shotaro Fujiwara, Takuto Ishida, Ryotaro Higuchi, Ayumu Kocha, Yo Muraoka, Yuki Nagashima, Hiroki Kocha, Atsuo Nakagawa, Yoshiyo Oguchi

**Affiliations:** ^1^ Department of Neuropsychiatry St. Marianna University School of Medicine Kawasaki Japan; ^2^ Department of Psychiatry Tokyo Metropolitan Matsuzawa Hospital Tokyo Japan; ^3^ Department of Internal Medicine Tokyo Metropolitan Matsuzawa Hospital Tokyo Japan

**Keywords:** drug‐resistant epilepsy, electroencephalography, epilepsy monitoring unit, postictal psychosis, psychosis, temporal lobe epilepsy

## Abstract

**Background:**

Postictal psychosis (PIP) is a serious complication that can follow seizure clusters, including those provoked during long‐term video‐electroencephalographic monitoring (LTVEM). Early detection and appropriate treatment of PIP are crucial to implement LTVEM safely. However, managing PIP becomes challenging when patients conceal symptoms, and psychotic symptoms are ambiguous.

**Case Presentation:**

A female patient in her 30s with drug‐resistant mesial temporal lobe epilepsy due to right hippocampal sclerosis underwent LTVEM at an epilepsy center. After seizure clusters occurred on hospital Days 2 and 3, she remained superficially stable apart from subtle psychotic symptoms beginning on hospital Day 4. Despite a thorough psychiatric assessment, she denied any psychotic experiences and was discharged as scheduled on hospital Day 7. Immediately after discharge, however, she displayed overt psychotic symptoms, necessitating emergency involuntary psychiatric admission. Her psychotic symptoms resolved within 16 days of psychiatric hospitalization. Upon recovery, she disclosed that she had concealed her symptoms during LTVEM because auditory command hallucinations had discouraged her from discussing the symptoms with her physicians, and her persecutory delusions, which were directed specifically toward the hospital and medical staff, had fostered a profound mistrust.

**Conclusion:**

The present case highlights a diagnostic pitfall caused by overlooking PIP occurring during LTVEM, particularly in patients in whom the psychotic content is directed against the hospital and medical staff. The absence of overt behavioral disturbances does not exclude PIP. Close multidisciplinary collaboration between epileptologists and psychiatrists is essential to ensure the patient's safety.

## BACKGROUND

Although long‐term video‐electroencephalographic monitoring (LTVEM) is valuable in evaluating epilepsy,^1^ it occasionally induces seizure clusters, a well‐established risk factor for postictal psychosis (PIP).^2^, ^3^, ^4^ PIP typically appears after a lucid interval of hours to days following the occurrence of seizure clusters^5^, ^6^ and has been reported in several observational studies of LTVEM with an estimated incidence of around 6%.^7^, ^8^, ^9^ PIP is clinically significant because it can lead to problematic behaviors such as self‐injury and aggression,^10^ and suicide attempts shortly after discharge from LTVEM have also been reported.^11^ Given the behavioral risks associated with PIP, some experts recommend monitoring for psychiatric symptoms within 3 days following seizure clusters.^6^ However, clinical practices vary across institutions,^1^ and there are currently no established criteria for evaluating PIP during LTVEM or for determining the observation period after LTVEM and the appropriate timing for discharge. Importantly, while PIP has attracted considerable attention for its lucid interval and severity of its symptoms, less attention has been paid to subtle or ambiguous psychiatric symptoms that emerge during LTVEM. Herein, we report a case of PIP that emerged during hospitalization for LTVEM. The patient concealed her symptoms due to psychosis. Overt psychotic symptoms became evident immediately after discharge, necessitating emergency psychiatric hospitalization.

## CASE PRESENTATION

A female patient in her 30s was admitted to an epilepsy center for LTVEM. She had a history of more than 20 years of mesial temporal lobe epilepsy due to right hippocampal sclerosis. Her past medical history included febrile seizures in early childhood but was otherwise unremarkable. Her family history was notable for schizophrenia in a maternal cousin. She had no history of substance use. Since adolescence, she had experienced focal impaired awareness seizures (FIAS) typically lasting about 1 min, during which she became unresponsive and exhibited automatisms predominantly involving the left upper limb with subsequent amnesia for the ictal events. Despite treatment with multiple antiseizure medications (ASMs), she continued to experience several FIAS per month during outpatient management (see Figure 1a for outpatient electroencephalogram [EEG]). She had declined epilepsy surgery 5 years earlier, despite her attending physician's recommendation. Over the preceding year, her seizures increased in frequency, occasionally occurring several times a day. Six months before the current admission, she had experienced an overt psychotic episode, which was diagnosed as PIP and required involuntary psychiatric hospitalization for approximately one month. At that time, cerebrospinal fluid (CSF) analysis revealed no abnormalities in cell count, protein, and glucose levels, and was negative for anti‐*N*‐methyl‐d‐aspartate receptor antibodies. Furthermore, three months before the current hospitalization, she was voluntarily admitted to a psychiatric unit for one month for irritability and emotional instability related to psychological distress caused by the recurrent seizures (see Figure 1b for the EEG during this hospitalization). No psychotic symptoms were observed during this admission.

Figure 1Longitudinal electroencephalographic recordings. Electroencephalographic recordings obtained (a) 14 months before long‐term video‐electroencephalographic monitoring (LTVEM) during stable outpatient follow‐up; (b) 3 months before LTVEM during voluntary psychiatric admission for rest due to recurrent seizures; (c, d) on Day 2 of LTVEM showing several focal impaired awareness seizures (FIAS); and (e) on Day 3 of psychiatric hospitalization when the patient exhibited a psychotic state. EEG, electroencephalography.

**Figure:**
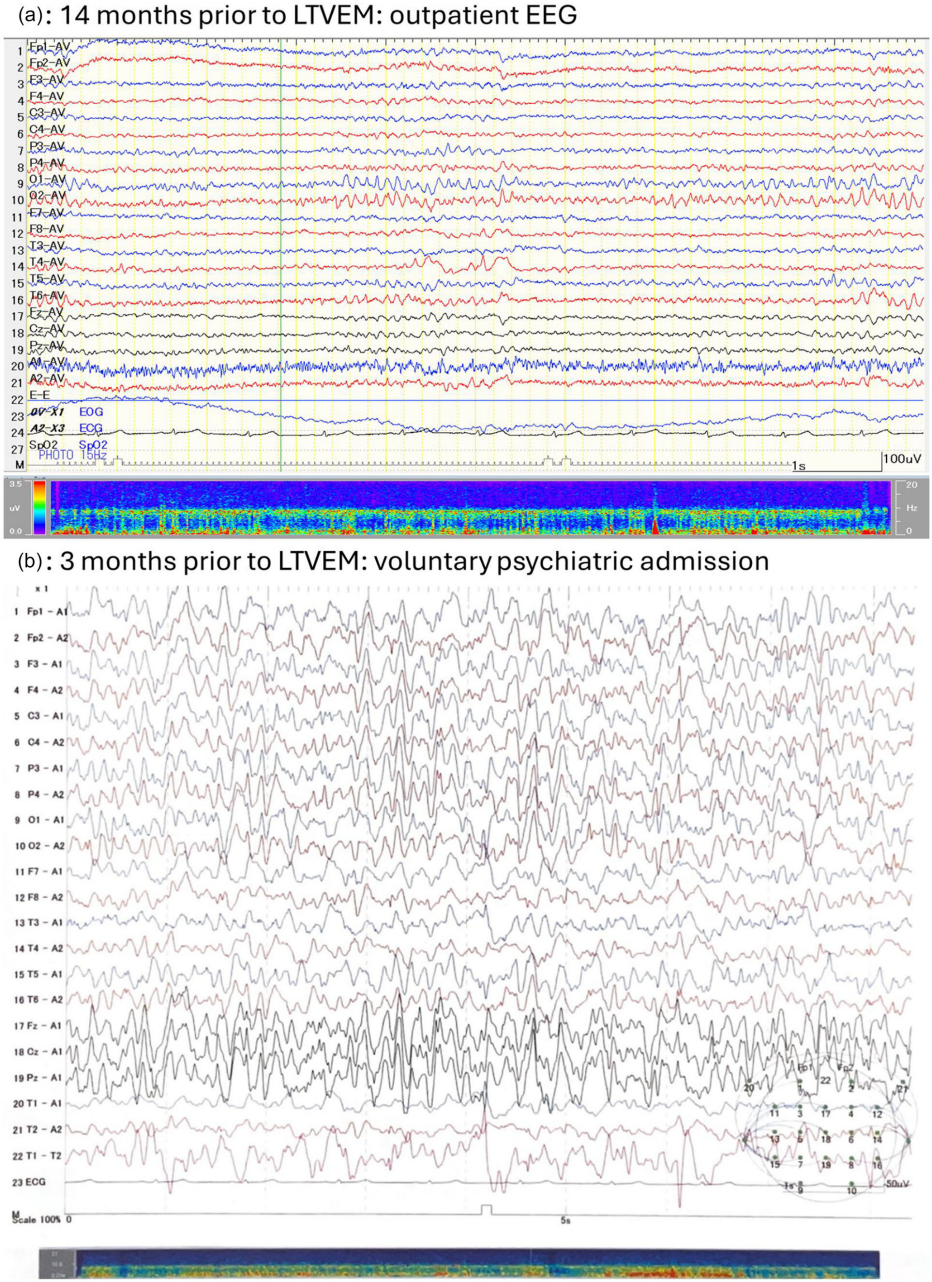

**Figure:**
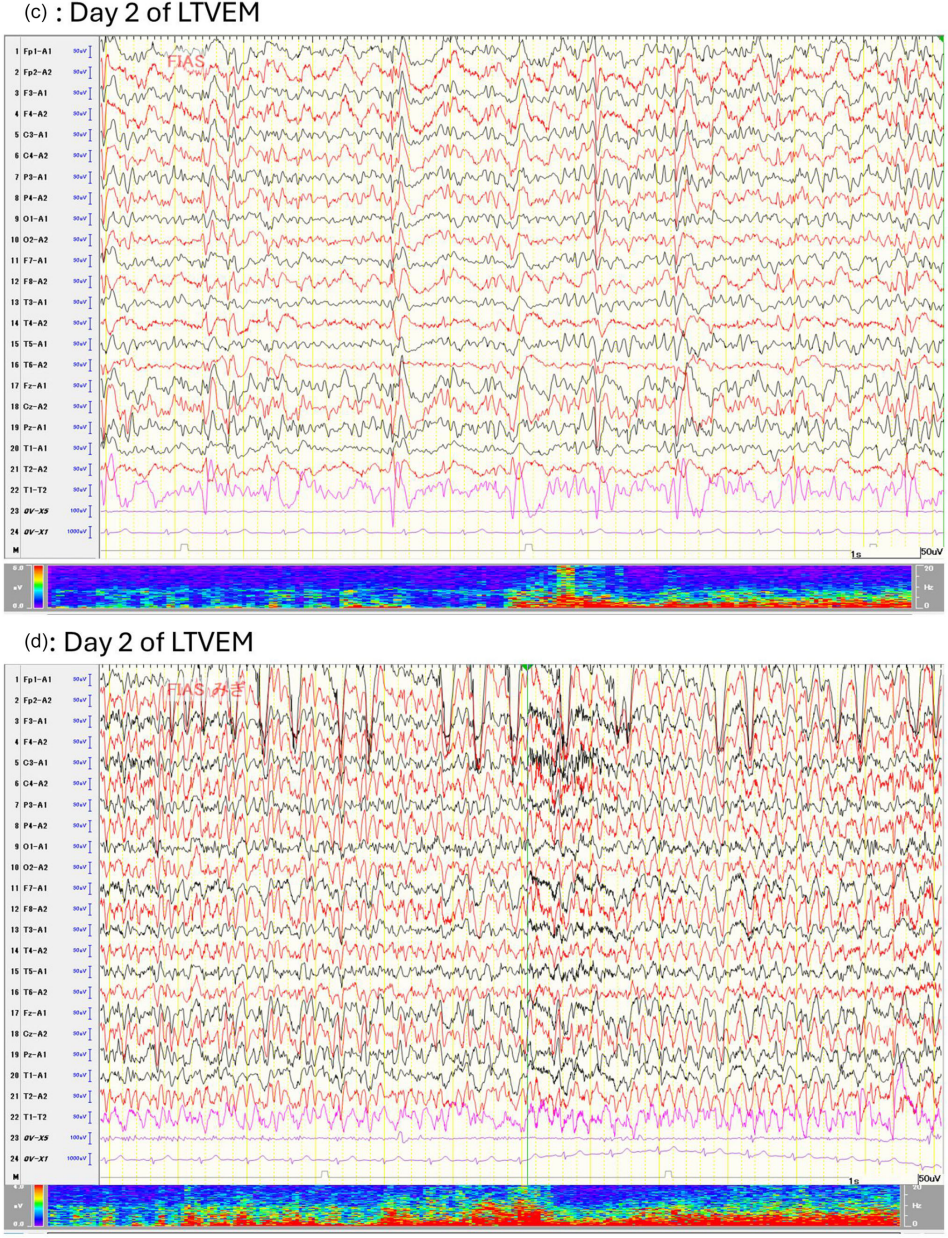

**Figure:**
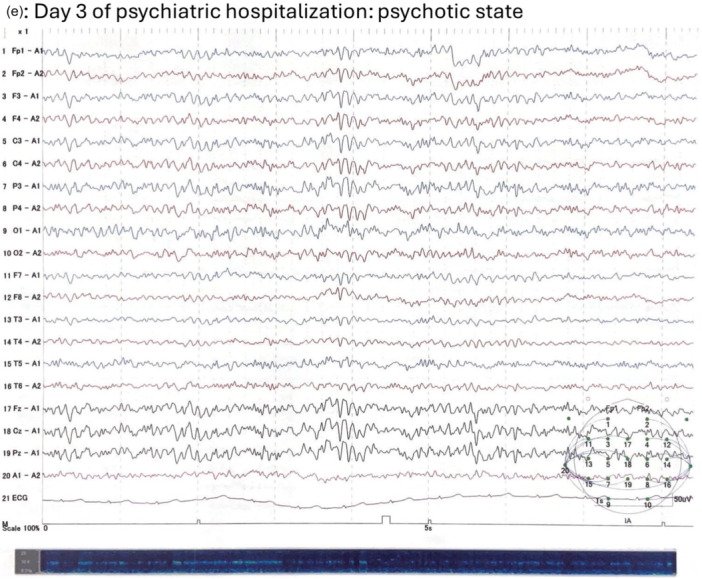

**Figure 2:**
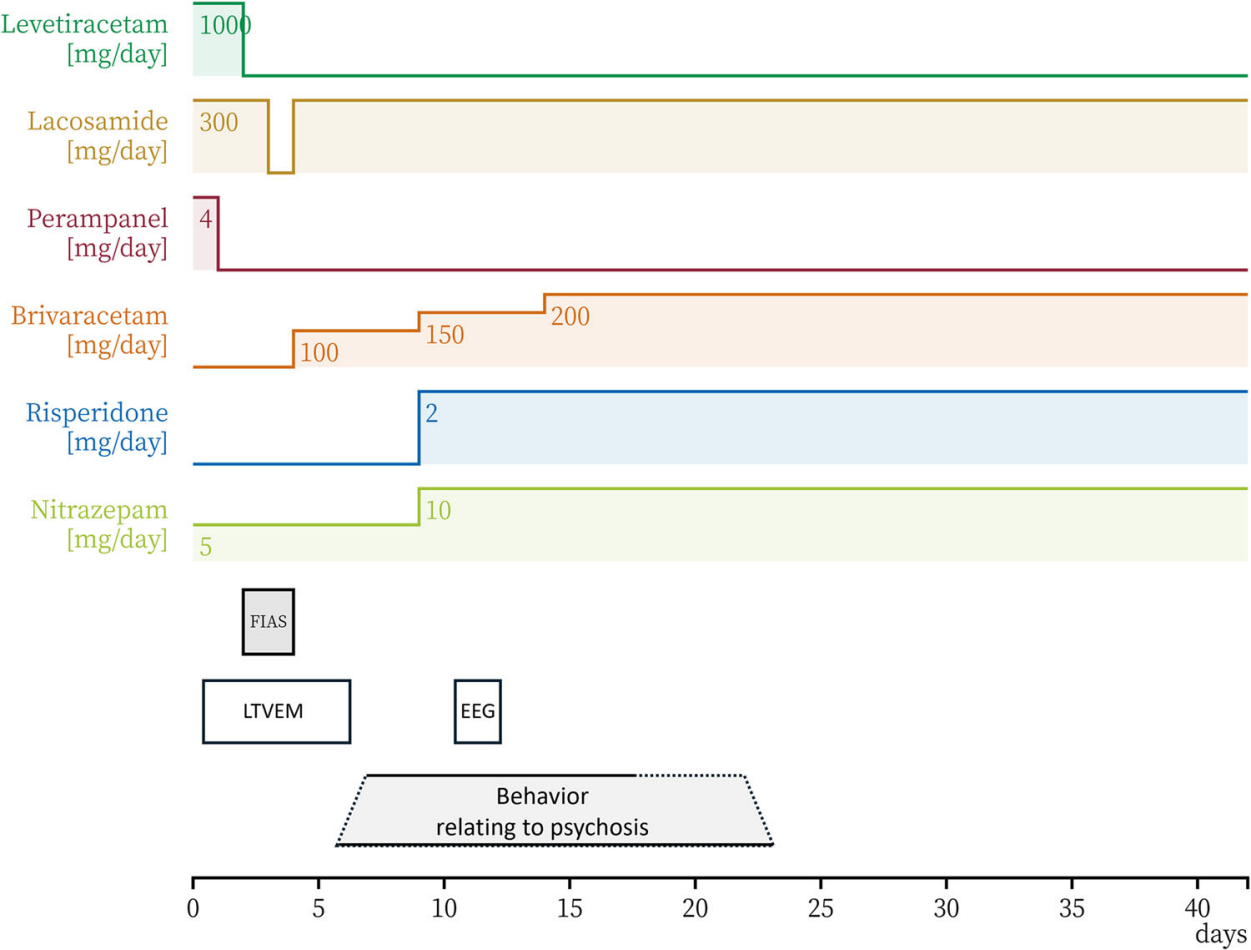
Clinical course. The timeline summarizes the clinical course, including seizure clusters, changes in medications, and psychiatric evaluations. EEG, electroencephalography; FIAS, focal impaired awareness seizures; LTVEM, long‐term video‐electroencephalographic monitoring.

She was referred to the epilepsy center for further epilepsy management and was followed by neurosurgeons and psychiatrists. Her outpatient ASMs had consisted of levetiracetam 1000 mg/day, lacosamide 300 mg/day, and perampanel 4 mg/day for 2 years. In preparation for surgery, she was admitted to the epilepsy center for LTVEM. The ASMs were tapered and discontinued upon admission (Figure 2). Several FIAS were recorded on hospital Days 2 and 3 (Figure 1c,d), after which no further seizures occurred. On hospital Day 2, she transiently complained of insomnia and nightmares. A consultation‐liaison psychiatric assessment on hospital Day 3 revealed no psychotic symptoms. On hospital Day 4, ASM administration was resumed. Given her history of irritability, the regimen was adjusted to brivaracetam 100 mg/day and lacosamide 300 mg/day. From that day onward, she reported intermittent auditory hallucinations, which she described as frightening whispering voices, and displayed irritability toward the staff, accusing them of “manipulating the atmospheric pressure” to distress her. She gradually became less forthcoming about her experiences and declined to discuss them. Her irritability was initially interpreted as a stress response similar to those she had shown in the past. A psychiatrist suspected psychosis, but the patient responded defensively, attributing her remarks to a dream she had while asleep and insisting she was “fine now” while repeatedly and strongly requesting discharge. Since she denied having symptoms and displayed no problematic behaviors such as self‐injury and aggression, involuntary psychiatric admission was not warranted, and she was discharged on hospital Day 7 as scheduled. However, during the discharge procedure, she made several statements that were apparently influenced by the hallucinations and persecutory delusions. Shortly after leaving the epilepsy unit, she returned in a tearful, disorganized, and restless state, pacing through the hospital and strongly insisting that she would be killed if she went outside. Given her anxious, tearful, and confused behavior and psychomotor agitation, the consultation‐liaison psychiatry team arranged immediate involuntary psychiatric admission.

**Figure 3:**
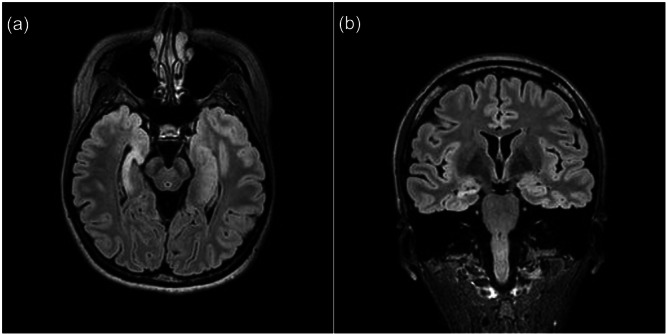
Brain magnetic resonance imaging findings. Fluid‐attenuated inversion recovery (FLAIR) images demonstrate right hippocampal atrophy with increased signal intensity, consistent with right hippocampal sclerosis, as shown in axial (a) and coronal (b) views.

On psychiatric admission, laboratory and neuroimaging findings ruled out organic factors other than known right hippocampal sclerosis (Figure 3). CSF analysis was not repeated during the present admission as a prior evaluation revealed no abnormalities. A standard EEG performed on psychiatric hospital Day 3 showed predominantly 10 Hz alpha activity with occasional theta‐range slow waves and no epileptiform discharges (Figure 1e). After psychiatric admission, she intermittently refused medical examinations, including additional EEG evaluations, along with food and medication. Standardized psychiatric evaluation, such as Positive and Negative Syndrome Scale (PANSS) or Brief Psychiatric Rating Scale (BPRS), could not be performed due to her refusal. She also made vague comments suggestive of self‐harm and persecutory delusions. Treatment with risperidone 2 mg/day and nitrazepam 10 mg/day was begun. Her psychotic symptoms gradually subsided, then resolved completely by psychiatric hospital Day 16. Upon recovery, she revealed that she had experienced multiple psychotic symptoms, including persecutory delusions that the hospital was harassing her and falsifying the LTVEM results, a poisoning delusion related to her food and medication, and threatening auditory and visual hallucinations, all of which she had concealed during the monitoring period. Furthermore, she reported experiencing auditory hallucinations which explicitly commanded her not to discuss her experiences with the physicians. She revealed that her concealment of symptoms, refusal of medical services, and marked reduction in food intake had been driven by these delusions and profound mistrust of the medical staff. After the patient's condition improved as her oral intake gradually recovered along with the arrangement of home health services, she was discharged on psychiatric hospital Day 35. Although risperidone was discontinued during the outpatient follow‐up, the patient's psychotic symptoms did not recur. She consented to surgery, for which she is currently being prepared.

## DISCUSSION AND CONCLUSION

The present case illustrates the difficulty of detecting PIP arising during LTVEM. Previous studies found that early PIP is often mistaken for a mood disorder^12^ and that psychiatric comorbidities are prevalent in epilepsy.^13^ The clinical features of this case, specifically, the lucid interval, rapid clinical improvement, and sustained remission after discontinuation of the antipsychotic medication, were consistent with PIP rather than with an exacerbation of baseline irritability, medication‐induced psychosis, or schizophrenia onset. However, the patient's PIP symptoms were initially mistaken for irritability stemming from psychological distress. This case highlights a critical pitfall in epilepsy care: in a patient with a psychiatric history, the warning signs of PIP may be dismissed as a typical complaint. Even seemingly mild or nonspecific psychiatric symptoms during LTVEM warrant careful evaluation for possible PIP.

This diagnostic confusion was further compounded by the symptoms of the patient's psychosis during LTVEM. Previous studies suggested that patients with PIP often do not spontaneously discuss their hallucinations or delusions, which clinicians may fail to recognize.^5^, ^14^ Moreover, in this case, the psychosis directed toward the staff and the hospital drove her to conceal her behavior and to refuse medical services. These behaviors in turn hindered a thorough assessment of the patient, leading to the underestimation of the severity of her condition. Since PIP develops during LTVEM in the hospital setting, the content of the patient's psychosis may be directed toward the hospital, medical staff, or treatment, potentially making clinical assessment more difficult than in the outpatient setting. These considerations highlight how a superficially stable presentation in the hospital setting can lead clinicians to overlook the presence of PIP and underestimate its potential behavioral risks.

When diagnostic uncertainty remains, the safety of LTVEM depends on multidisciplinary evaluation. While neurologists have greater knowledge of PIP than psychiatrists in one survey,^15^ the non‐psychiatrist's ability to diagnose new‐onset psychosis is limited.^16^ Taken together, these points underscore the need for multidisciplinary assessment, specifically with early involvement of consultation‐liaison psychiatry. For patients at high risk of PIP, having a psychiatrist assess the mental status at baseline allows for the early detection of PIP from subtle deviations, such as reducing oral intake, becoming less forthcoming about their experiences, reacting defensively to psychiatric inquiries, or requesting discharge despite the ongoing examination plan. When psychiatric symptoms are subtle and clinical judgment is uncertain, close monitoring is warranted even in the absence of overt behavioral disturbances. If hospitalization for observation is not feasible, a structured outpatient follow‐up system should be established to allow rapid intervention in case of symptom escalation.

Although this report discusses only a single patient, it has important implications for the management of PIP occurring during LTVEM. Given that PIP occurring in the hospital setting can drive patients to conceal their symptoms, multidisciplinary evaluation throughout LTVEM is required to ensure the patient's safety. Clinicians should remain vigilant for subtle indicators of concealment, such as guardedness, evasion, and defensive reactions. A stepwise, observational process involving monitoring of the patient's activity within the ward, supervised passes and short leaves, and a temporary transfer to a psychiatric unit can be beneficial. A questionnaire administered in the outpatient setting^14^ might serve to screen for PIP or aid clinical judgment, but the use of such a questionnaire during LTVEM requires closer study. Further evidence is needed to establish a comprehensive, multidimensional protocol for the psychiatric evaluation and risk management of patients during and after LTVEM, as well as to better characterize the prevalence and clinical implications of symptom concealment in this population.

## AUTHOR CONTRIBUTIONS

Shotaro Fujiwara, Ryotaro Higuchi, Ayumu Kocha, Yo Muraoka, and Yuki Nagashima acquired the case data. Shotaro Fujiwara drafted the manuscript with support from Takuto Ishida and Atsuo Nakagawa. Hiroki Kocha and Atsuo Nakagawa supervised the work and critically revised the manuscript. Yoshiyo Oguchi provided overall supervision of the project. All authors contributed to the manuscript and approved the final version.

## CONFLICT OF INTEREST STATEMENT

The authors declare no conflicts of interest.

## ETHICS APPROVAL STATEMENT

This case report was conducted in accordance with ethical guidelines for case reports of the Japanese Society of Psychiatry and Neurology. Written consent from the patient was obtained for the publication of the details of this case.

## PATIENT CONSENT STATEMENT

Written consent from the patient was obtained for the publication of the details of this case.

## CLINICAL TRIAL REGISTRATION

N/A.

## Data Availability

The data that support the findings of this study are available on request from the corresponding author. The data are not publicly available due to privacy or ethical restrictions.
